# Global consensus statement on simulation-based practice in healthcare

**DOI:** 10.1186/s41077-024-00288-1

**Published:** 2024-05-21

**Authors:** Cristina Diaz-Navarro, Robert Armstrong, Matthew Charnetski, Kirsty J. Freeman, Sabrina Koh, Gabriel Reedy, Jayne Smitten, Pier Luigi Ingrassia, Francisco Maio Matos, Barry Issenberg

**Affiliations:** 1 Perioperative Care, Cardiff and Vale University Health Board, Wales, UK; 2 School of Health Professions, Eastern Virginia Medical School, Norfolk, VA USA; 3 Simulation-Based Education and Research, Dartmouth Health, Lebanon, NH USA; 4 Geisel School of Medicine, Dartmouth College, Hanover, NH USA; 5 School of Health Professions Education, Maastricht University, Maastricht, Netherlands; 6 The Rural Clinical School of Western Australia, The University of Western Australia, Perth, Australia; 7 SingHealth Duke-NUS Institute of Medical Simulation, SingHealth Academy, Singapore, Singapore; 8 Nursing Education and Development, Sengkang General Hospital, Singapore, Singapore; 9 Centre for Education, Faculty of Life Sciences and Medicine, King’s College London, London, UK; 10 School of Nursing, Hawai’i Pacific University, Honolulu, Hawaii USA; 11 Centro Professionale Sociosanitario MedicoTecnico, Lugano, Switzerland; 12 Hospitais da Universidade de Coimbra, Coimbra, Portugal; 13 Faculty of Medicine, Coimbra University, FMUC, Coimbra, Portugal; 14 Clinical Academic Center of Coimbra, CACC, Coimbra, Portugal; 15 University of Miami Miller School of Medicine, Miami, FL USA

## Abstract

Simulation plays a pivotal role in addressing universal healthcare challenges, reducing education inequities, and improving mortality, morbidity and patient experiences. It enhances healthcare processes and systems, contributing significantly to the development of a safety culture within organizations. It has proven to be cost-effective and successful in enhancing team performance, fostering workforce resilience and improving patient outcomes.

Through an international collaborative effort, an iterative consultation process was conducted with 50 societies operating across 67 countries within six continents. This process revealed common healthcare challenges and simulation practices worldwide. The intended audience for this statement includes policymakers, healthcare organization leaders, health education institutions, and simulation practitioners. It aims to establish a consensus on the key priorities for the broad adoption of exemplary simulation practice that benefits patients and healthcare workforces globally.

**Key recommendations** Advocating for the benefits that simulation provides to patients, staff and organizations is crucial, as well as promoting its adoption and integration into daily learning and practice throughout the healthcare spectrum. Low-cost, high-impact simulation methods should be leveraged to expand global accessibility and integrate into system improvement processes as well as undergraduate and postgraduate curricula. Support at institutional and governmental level is essential, necessitating a unified and concerted approach in terms of political, strategic and financial commitment.

It is imperative that simulation is used appropriately, employing evidence-based quality assurance approaches that adhere to recognized standards of best practice. These standards include faculty development, evaluation, accrediting, credentialing, and certification.

We must endeavor to provide equitable and sustainable access to high-quality, contextually relevant simulation-based learning opportunities, firmly upholding the principles of equity, diversity and inclusion. This should be complemented with a renewed emphasis on research and scholarship in this field.

**Call for action** We urge policymakers and leaders to formally acknowledge and embrace the benefits of simulation in healthcare practice and education. This includes a commitment to sustained support and a mandate for the application of simulation within education, training, and clinical environments.

We advocate for healthcare systems and education institutions to commit themselves to the goal of high-quality healthcare and improved patient outcomes. This commitment should encompass the promotion and resource support of simulation-based learning opportunities for individuals and interprofessional teams throughout all stages and levels of a caregiver’s career, in alignment with best practice standards.

We call upon simulation practitioners to champion healthcare simulation as an indispensable learning tool, adhere to best practice standards, maintain a commitment to lifelong learning, and persist in their fervent advocacy for patient safety.

This statement, the result of an international collaborative effort, aims to establish a consensus on the key priorities for the broad adoption of exemplary simulation practice that benefits patients and healthcare workforces globally.

## Introduction

Healthcare simulation is “a technique that creates a situation or environment to allow persons to experience a representation of a real event for the purpose of practice, learning, evaluation, testing, or to gain understanding of systems or human actions” [1]. Beyond education and training, it plays a pivotal role in optimizing healthcare systems, thereby enhancing healthcare delivery, promoting staff well-being and improving patient safety and outcomes [2, 3].

The dynamics of knowledge sharing and interaction within healthcare communities have evolved significantly in recent years, driven by exponential access to virtual platforms [4]. This transformation has heightened awareness of the diverse landscape of simulation practices in healthcare worldwide. This underscores the need for a unified global position on needs, solutions and priorities. A consensus-driven process enabled thoughtful consideration of key variations in conditions and practices, ultimately fostering global alignment on future directions [5].

This collaboration, facilitated by the Society for Simulation in Europe (SESAM) and the Society for Simulation in Healthcare (SSH), aims to articulate a global perspective on the current scope of simulation-based practice and gain consensus on future guidance. It emphasizes the crucial role of simulation in enhancing healthcare practices and education, as well as its far-reaching impact. The resulting recommendations aim to promote widespread adoption of simulation practices, benefiting patients and healthcare workforces globally. Policymakers, healthcare organization leaders, health education institutions, and simulation practitioners are the intended recipients of this valuable insight.

A global perspective has been rigorously crafted through extensive and iterative consultation. Representatives from 50 national and international simulation societies and networks distributed across 67 countries were actively engaged in this collaborative effort.

The chosen themes for discussion encompassed key healthcare challenges, the current landscape of simulation use, and ethical considerations within simulation practice. Prior to virtual encounters, relevant questions were emailed, and initial input was thoughtfully provided during structured online meetings during November 2023. All individual contributions were inclusively aggregated through an implicit approach, and emerging themes were summarized into narrative statements and tables.

Consensus on the identified themes was attained during face-to-face meetings held in January 2024. Subsequently, key areas were prioritized through an online survey. The initial draft of this document was produced in February 2024, and then shared with all contributors for peer review. Out of 24 responses, every comment received was thoroughly considered and significantly contributed to the final production of this document.

### Current state of simulation practice in healthcare

Healthcare simulation finds application across the spectrum of health and care, involving all clinical disciplines and allied professions including dental, mental health and social care. Within this context, simulation practice serves educational and non-pedagogical uses. It encompasses activities such as device, process, system testing, system integration, quality improvement, research and innovative approaches [2, 3]. Contributions obtained during consultation have highlighted that simulation serves as an adjunct to therapeutic interventions. It is employed in diverse situations, including complex case-by-case surgical planning, aiding pain management during labor, supporting cognitive behavioral therapy in mental health settings and facilitating the training of social skills for autistic patients.

Furthermore, there has been an exponential integration of simulation approaches into quality improvement and patient safety efforts within healthcare teams, departments, and organizations.

Notably, simulation transcends the confines of healthcare systems. It emerges as an excellent public engagement tool and plays a crucial role in multi-agency team preparedness for disaster management.

The value of healthcare simulation is vast and encompasses a wide array of tools and practices. These include, but are not limited to part task trainers, patient simulators (i.e. manikins), cadaveric simulation, and standardized patients or simulated participants portraying patients, relatives, by-standers, and healthcare colleagues. It also includes telesimulation, computer-based simulation, tabletop exercises, data modeling and extended realities including augmented reality, virtual reality, mixed reality, and haptic feedback models. As a community of practice, we provide unique opportunities to learn, rehearse and enhance the wide array of capabilities required to care for all patients, from carrying out simple procedures to managing rare and life threatening situations. We nurture the development of patient-centered communication skills, situational awareness, decision making, team working, leadership and other essential professional behaviors. We continue to innovate and adapt, developing new initiatives according to emerging needs, such as the delivery of packaged simulation materials to students in order to facilitate remote learning during the COVID-19 pandemic. Likewise, this creativity is crucial when supporting healthcare learning in low resource and rural settings, with an increasing use of telesimulation and “pack-and-go” equipment.

Contributions received during the consultation process highlighted the universal challenge of disparities in access to simulation education and resources across geographical areas and socio-economic contexts as well as between different institutions and specialties. Different professions continue to learn within isolated silos, with insufficient opportunities for interprofessional education, particularly in clinical environments. These inequities result in uneven development of competencies, and are encountered at both undergraduate and postgraduate levels, revealing a clear imperative to integrate simulation into healthcare curricula and into everyday learning opportunities within healthcare organizations.

Additional challenges reported include insufficient standardization of simulation training programmes and inadequate quality assurance of practices, particularly related to assessment and faculty development. A novel challenge arises from the impact of the COVID-19 pandemic on student development. Not only have students experienced reduced exposure to clinical environments, but also to in-person simulation. Consequently, they might initially perceive immersive settings as intimidating.

However, simulation offers global opportunities to support healthcare capabilities. For instance, it aids in preparing health professionals as they enter the workforce. Additionally, simulation helps mitigate skill degradation, especially in the context of high-risk low-frequency situations such as cardiopulmonary resuscitation performance by healthcare personnel or bystanders.

### Overcoming healthcare challenges

The consultation process has identified healthcare challenges with a global reach (Table 1). These challenges encompass significant inequalities in access to healthcare and safety culture, extending to education and training for healthcare professions at undergraduate and postgraduate levels. Financial constraints contribute to disparities in healthcare and education, with lower income countries experiencing the most pronounced effects. The consequences of inadequate funding and resource allocation reverberate throughout healthcare systems and culture, limiting the onboarding, upskilling, and continuing education of healthcare staff and teams. Ultimately, these challenges have a negative impact on the workforce, patients, and societies at large.

**Table 1:** Global healthcare challenges identified during consensus consultation phase

Theme	Subtheme	Challenges
Healthcare systems	System design	Systemic/societal issues impact healthcare Short term planning with regard to training and workforce Insufficient workforce resource for patient volume and burden-of-care Political pressures
	Distribution of care	Vast global inequities in healthcare quality and access as well as in training for healthcare professions Variable patient care distribution across hospitals, primary care and rural care Redistribution of healthcare also impacts social care Primary care and community care sector unable to cope with demand Increased number of referrals from primary care to specialist hospitals Lack of patient-centered collaboration between community health services and hospitals
	Demand on services	Long waiting lists and extended waiting times in Emergency Departments Hospitals continually at over-capacity, with subsequent risk to patients Long waiting times for diagnosis in pediatric/young adult mental health Increasing amounts of mental health issues in general and specifically after covid Need for wellness plans in geriatric and younger age groups: teenagers and children Increasing complexity of the case mixes Burden of: - chronic non-communicable diseases - infectious diseases associated with vectors in tropical regions or those with sanitation problems - injuries related to general trauma and violence - Trauma victims and mass incidents
	Funding	Uneven distribution of budget across care areas Financial limitations and budget cuts Financial restrictions impact on continued education of healthcare staff, in particular IPE - Inadequate preparation of unqualified healthcare workers - Insufficient funding for simulation programs - Sustainability of simulation programs
	Organizational culture and leadership	Inadequate leadership Low interest in promoting best practice Political pressures affection prioritization Insufficient investment in staff development (CPD) Low priority given to educational activities Inadequate support for staff with neurodivergence
	Safety culture	Blame culture Work as imagined v work as done Loopholes in patient safety
	Education	Insufficient opportunities for interprofessional education in healthcare environments Insufficient time allocation for staff to attend simulation training opportunities Inadequate recognition of the value of simulation Inadequate workforce promotion pathways when working in simulation Lack of instructors/trainers/preceptors in healthcare settings Insufficient number of training positions In some settings, low volume of patients may be insufficient to maintain clinical competencies Progressive limitation in real world clinical training at undergraduate and postgraduate levels
	Technology	Transformative consequences for the future of healthcare Lack of regulation for its introduction in healthcare Variable digital skills/readiness within workforce Slow adoption curve for change and technology in healthcare Need for innovative data management and interpretation methods, including modeling, analysis and simulation
Staff	Burnout	Deep gaps in pay Low pay, low morale, insufficient study leave Healthcare work is progressively less valued by the wider society Poor work-life balance Clinicians’ use of unpaid time to teach and learn Inadequate preparation of new healthcare professionals for the reality of their working life Violence at the workplace
	Availability	Difficulties in staff recruitment and retention Staff relocation International impact of migration of healthcare providers (emigration leads to skill loss, immigration requires credentialing and onboarding) Aging workforce
	Healthcare practice	Changing scopes of practice Defensive medical practice Inadequate skills in interruption management Limited exposure to healthcare environments during pandemic in undergraduate education and consequences in care Differences in communication, application of knowledge and practice
Patients	Demographics	Aging population with increasing healthcare needs Intercultural context, ethnicity differences and disproportions in health Socioeconomic differences and their impact Geographical inequity
	Interaction with services	Changing expectations Patient empowerment: involvement in decision-making, access to care records and results
	Outcomes	High mortality and morbidity rates Maternal-perinatal morbidity Young adults falling through the cracks of society (unemployed, unschooled etc.) Malpractice/iatrogenic injury/complication/error related litigations

The role of simulated practice in overcoming these challenges is paramount. For example, simulation has demonstrated a positive impact in reducing education inequities, leading to reductions in mortality and morbidity in low-resource areas [6, 7]. In addition, it supports improvements in patient experiences [8]. Simulation interventions contribute to the optimization of healthcare processes and systems, and to organizational safety culture [9–11]. They have proven to be cost-effective and successful in enhancing team performance [12], while also fostering workforce well-being and resilience [13, 14]. It is indisputable that simulation improves healthcare practices, such as central venous catheter placements, leading to a decrease in related infections, and improving patient outcomes [15, 16].

Simulation can help adapt to the changing demands on healthcare systems, for instance preparing clinicians to manage complexity [17], and supports the development of skills for health and social care professionals in caring for an aging population [18]. Additionally, it improves team performance in managing trauma victims and mass casualty disasters [19, 20].

Other evolving challenges include the ongoing transformation of healthcare practice and education from technological developments. While there is little regulation regarding their introduction, staff digital skills often lag behind, impacting the adoption curve for technological changes in healthcare settings. Regardless, the increasing volume of health data necessitates innovative methods for management and interpretation, including the use of modeling, analysis and simulation [20].

### Ethical considerations

Ethical considerations focus on issues that may be interpreted as “morally right or wrong, just or unjust” [21]. These considerations help to ensure that all individuals involved in healthcare simulation are treated and treat others with integrity, respect, empathy, and compassion.

The consultation process revealed a wide array of ethical considerations of importance to the global healthcare community (Table 2). A foundational requirement is to promote equitable access to high-quality healthcare, including dental, mental health, and social care. Simulation complements the development and refinement of caregiving skills, which are essential for all practitioners to deliver the excellent healthcare that every patient deserves. Therefore, global availability of healthcare simulation is an ethical imperative. Concurrently, opportunities for simulation faculty development must be identified globally, with consideration of their affordability in low-resource settings.

**Table 2:** Ethical considerations in simulation in healthcare provided during the consultation process

Theme	Considerations
Equity of access	High quality healthcare including dental, mental health and social care, should be universally accessible - Workforce burnout globally - Support for staff in difficulty Equitable access to education - Identification of educational requirements: “Learners don’t know what they don’t know” - Prioritization of time and staff investment into educational activities - Cost of training and availability of resources - Identification of pathways for resource sharing (e.g. faculty exchanges or “pack-and-go” approaches) - Affordability of faculty development in low resource settings - Equitable fees for access to international resources
Safety culture	Safety to learners and patients (psychological and physical) - Balancing safety of learners and patients when red flags are demonstrated Protection of confidential patient information Psychological safety in debriefing - Supported by faculty training - Confidentiality - Support learner vulnerabilities Avoidance of “shame and blame” culture, both in simulation and healthcare settings
Acknowledgement of diversity equity and inclusion (DEI / EDI)	Cultural differences Silo practices Multicultural societies Complex cultural variation within one area Ethnic minorities Equitable partnership between - health profession - first responders and care providers - national and international collaborations
Emerging technologies	Use of new technologies and benefits v risks: - Digital twins - Computer modeling and data generation to support research and analysis - AI as an enabler, a tool to deal with complex tasks
Sustainability	Carbon footprint of simulation Workforce sustainability in education and healthcare Sustainability of simulation programs

As with all relevant tools, medicines, and interventions, healthcare simulation must be employed ethically. This includes a commitment to and adherence with common guidelines, such as the standards produced by the International Nursing Association of Clinical Simulation and Learning (INACSL), the Association for Simulated Practice in Healthcare (ASPiH) and the Association of Standardized Patient Educators (ASPE), as well as the Healthcare Simulationist Code of Ethics [22–25].

Encouraging and fostering a shared safety culture mindset is critical. This ensures the psychological and physical safety of all participants, protects personal and patient information, and removes “blame and shame” feedback from learning and operational culture [23]. Furthermore, learners should be supported in the process of experiential learning with integrity and transparency, and in accordance with best practices [25].

Diversity, equity, inclusion, and accessibility principles are essential in simulation and healthcare practice [26]. By intentionally integrating these principles, we create a more culturally competent and responsive environment. Teams, institutions, and broader healthcare contexts must actively manage complex cultural relationships. Additionally, fostering equitable collaborative partnerships across all levels of care and education is crucial.

While incorporating advancing technologies into healthcare simulation is valuable, it is equally important to proceed judiciously. We should minimize potential unforeseen negative learning outcomes by carefully evaluating and implementing these innovations.

As fellow stewards of the planet, we bear a collective responsibility. Encouraging a shared mindset of sustainability and conservation is imperative [27].

### Recommendations

The global consultation process has yielded several key themes for recommendations (Table 3). The subsequent recommendations aim to provide alignment and direction to simulation professionals, healthcare systems, healthcare education institutions, and global leaders.

**Table 3:** Themes for recommendations captured during the consultation process

Theme	Considerations
Adoption into everyday learning and practice	Advocate for the use of simulation in patient safety initiatives Advance the institutional use of simulation for the improvement of adaptation to new challenges, productivity and burn out prevention Promote the “Return on experience”: the value of well trained healthcare providers Low cost simulation options may provide suitable and accessible learning experiences Foster the use of simulation throughout the training continuum, including CME and certification/recertification Simulation facilitation skills can support everyday training in any setting Emphasize the need for interprofessional education and team training Prioritize participant and patient safety Nurture simulation literacy Engage in and encourage collaboration
Integration into curricula	Harmonization between undergraduate and postgraduate simulation curricula Development of interprofessional learning opportunities Integration should follow a collaborative, wise and prudent approach, following best practice Simulation curricula should be aligned
Quality Assurance (QA)	Simulation is only good if used correctly: promote and develop standards for best practice Faculty development is crucial Evaluate all simulation activity Promote the inclusion of the patient’s perspective Embed risk assessment in simulation-based practice Certification of simulation devices Consider QA during the development and early adoption of emerging technologies QA is the responsibility all simulation practitioners, managers, healthcare and education organizations and policy makers Develop Internationally agreed frameworks for mapping of socio-cognitive skills Develop and adopt practical and standardized quality assurance tools Develop quality assured approaches for accreditation, credentialing, certification/recertification
Equitable access	High quality simulation learning opportunities should be equitably accessible and appropriate to each context and learning needs High impact low cost simulation and telesimulation may facilitate accessibility - Across the spectrum of professions and practice - To low income areas - To rural or remote environments Develop outreach programmes Facilitate participation of individuals from low income areas to international learning events Commitment to equity, diversity and inclusion both within and via simulation
Fostering research and scholarship	Promotion of simulation-based research focused on patient outcomes Simulation curricular alignment could facilitate educational research
Collaboration	Collaborate with patient safety associations or institutions and quality improvement agencies Foster networking
Societal expectations	“Your first attempt should never be on a live patient” “Everybody should be a master” “Failure is not an option” Pressure to perform Public engagement: - Potential of simulation to help shift patient perspectives, responsibilities and behaviors - Sharing what we do everyday with the public
Sustainability	Environmental sustainability of simulation facilities, considering structural designs, resource consumption and reutilisation, environmentally friendly materials, and awareness of the carbon footprint - telemedicine/telesimulation might support diminishing our carbon footprint Sustainability of high quality simulation programmes, simulation staff and the wider workforce across the globe
Enablers	Political and strategic support Practical support including staffing and other resources Societies Networks
Policy	Institutional and government level Appropriate prioritization of investment in educational resources (people over technology) Support to the sustainability of simulation facilities and programmes Explicit guidance for substitution of clinical placements with simulation practice (proportion and quality assurance)

First and foremost, it is necessary to advocate for the benefits that simulation brings to patients, staff and organizations. Promoting its adoption and integration into daily learning and practice across the entire spectrum of healthcare is essential. Beyond enhancing care providers’ and teams’ performances, simulation can also empower patients by providing new perspectives and fostering necessary responsibilities and beneficial behaviors, ultimately leading to improved patient outcomes.

Political, strategic and financial support at an institutional and governmental level is vital. Ensuring the sustainability of simulation facilities, programmes, and workforce requires concerted efforts and commitment.

Exploring low-cost high-impact simulation methods can expand its use throughout the training continuum. Particularly in interprofessional learning contexts, such approaches can be transformative. Simultaneously, integrating simulation into system improvement processes as well as undergraduate and postgraduate curricula, should follow a collaborative, prudent approach based on best practices.

There is a global agreement that simulation must be used appropriately. To enhance its effectiveness, we propose several key strategies:

- Development and use of evidence-based tools to ensure the quality of healthcare practice. These tools should be aligned with recognized standards of best practice and evolve alongside simulation methodologies.
- Invest in faculty development to enhance their expertise in simulation practice.
- Rigorously evaluate all simulation activities to maintain quality standards.
- Establish quality-assured approaches for accrediting, credentialing, and certifying (and recertifying) simulation programs and practitioners
- Provide equitable access to high-quality, contextually relevant simulation-based learning opportunities. To achieve this, it is critical to cultivate the support necessary to ensure consistent resourcing for healthcare simulation.
- Leverage telesimulation and virtual approaches to facilitate accessibility across the spectrum of professions and practice, including rural, remote and low-income areas.
- Uphold the principles of equity, diversity and inclusion both within and via simulation.
- Be mindful of the environmental impact of simulation activities.
- Encourage a renewed emphasis on research and scholarship in order to progress as a community of practice. Focus on simulation-specific initiatives and explore novel ways to integrate simulation into broader healthcare research and innovation.

### Call for action

Healthcare simulation serves a greater purpose beyond its own existence. Its mission is to elevate the performance of healthcare providers, teams and systems, ultimately leading to improved health outcomes for patients, communities, and societies. To achieve this transformative impact, it will require a concerted effort by leaders and policymakers, healthcare systems, healthcare education institutions, and simulation practitioners to promote and enhance this critical capability as a means of improving patient outcomes across the globe.

To this end, we propose several key actions:

We propose that policymakers and leaders formally acknowledge and embrace the benefits of simulation in healthcare practice and education, which ultimately enhance patient outcomes by:

- Committing sustained resources to simulation.
- Mandating the use of simulation within education, training, and clinical environments.
- Being explicit in how simulated experiences may augment or replace clinical experiences for learners in residency and pre-licensure status.

We recommend that healthcare systems and healthcare education institutions commit to the goal of high-quality healthcare and improved patient outcomes by:

- Promoting healthcare simulation as a critical and necessary learning tool throughout all phases and levels of a caregiver’s career.
- Providing the necessary resourcing for healthcare simulation, including staff, equipment, space, and curricular context.
- Using healthcare simulation to create interprofessional education and training opportunities.
- Fostering and adhering to healthcare simulation best practice standards.
- Cultivating simulation-capable faculty and mentors.

We call on simulation practitioners to:

- Promote healthcare simulation as a critical learning tool.
- Adhere to best practice standards.
- Perform to the highest levels of personal integrity and ethical behavior.
- Commit to lifelong learning.
- Persist in their fervent advocacy for patient safety.

We hope that this global statement contributes to increasing the visibility of simulation in healthcare, and guides the coordination of simulation and healthcare strategies and policies worldwide.

## References

[1] Healthcare Simulation Dictionary –Second Edition. Rockville, MD: Agency for Healthcare Research and Quality; September 2020. AHRQ Publication No. 20–0019. 10.23970/simulationv2

[2] Nickson CP, Petrosoniak A, Barwick S, Brazil V (2021). Translational simulation: from description to action. Adv Simul.

[3] Weldon SM, Buttery AG, Spearpoint K, Kneebone R. Transformative forms of simulation in health care-the seven simulation-based'I's: a concept taxonomy review of the literature. Int J Healthcare Simul. 2023:1–3.

[4] Slater BJ, Kashyap MV, Calkins CM, Powell D, Rothstein DH, Clifton M, Pandya S (2022). Global dissemination of knowledge through virtual platforms: Reflections and recommendations from APSA/IPEG. J Pediatr Surg.

[5] Murphy MK, Black NA, Lamping DL, McKee CM, Sanderson CF, Askham J, Marteau T (1998). Consensus development methods, and their use in clinical guideline development. Health Technol Assessment (Winchester, England).

[6] Mduma E, Ersdal H, Svensen E, Kidanto H, Auestad B, Perlman J (2015). Frequent brief on-site simulation training and reduction in 24-h neonatal mortality—an educational intervention study. Resuscitation.

[7] Nelissen E, Ersdal H, Mduma E, Evjen-Olsen B, Twisk J, Broerse J, van Roosmalen J, Stekelenburg J (2017). Clinical performance and patient outcome after simulation-based training in prevention and management of postpartum haemorrhage: an educational intervention study in a low-resource setting. BMC Pregnancy Childbirth.

[8] van Tetering AA, Ntuyo P, Martens RP, Winter N, Byamugisha J, Oei SG, Fransen AF, van der Hout-van MB. Simulation-based training in emergency obstetric care in sub-Saharan and Central Africa: a scoping review. Ann Global Health. 2023;89(1). 10.5334/aogh.3891 PMC10540704 37780839

[9] Ajmi SC, Advani R, Fjetland L, Kurz KD, Lindner T, Qvindesland SA, Ersdal H, Goyal M, Kvaløy JT, Kurz M (2019). Reducing door-to-needle times in stroke thrombolysis to 13 min through protocol revision and simulation training: a quality improvement project in a Norwegian stroke centre. BMJ Qual Saf.

[10] Brazil V, Purdy E, Bajaj K. Simulation as an improvement technique. Cambridge University Press; 2023.

[11] Schram A, Paltved C, Lindhard MS, Kjaergaard-Andersen G, Jensen HI, Kristensen S (2022). Patient safety culture improvements depend on basic healthcare education: a longitudinal simulation-based intervention study at two Danish hospitals. BMJ Open Quality.

[12] Ajmi SC, Kurz MW, Ersdal H, Lindner T, Goyal M, Issenberg SB, Vossius C (2022). Cost-effectiveness of a quality improvement project, including simulation-based training, on reducing door-to-needle times in stroke thrombolysis. BMJ Qual Saf.

[13] Sullivan J, Al-Marri A, Almomani E, Mathias J (2021). The impact of simulation-based education on nurses’ perceived predeployment anxiety during the COVID-19 pandemic within the cultural context of a middle eastern country. J Med Educ Curric Dev.

[14] Madrigano J, Chandra A, Costigan T, Acosta JD (2017). Beyond disaster preparedness: Building a resilience-oriented workforce for the future. Int J Environ Res Public Health.

[15] Barsuk JH, Cohen ER, Potts S, Demo H, Gupta S, Feinglass J, McGaghie WC, Wayne DB (2014). Dissemination of a simulation-based mastery learning intervention reduces central line-associated bloodstream infections. BMJ Qual Saf.

[16] Barsuk JH, Cohen ER, Feinglass J, McGaghie WC, Wayne DB (2009). Use of simulation-based education to reduce catheter-related bloodstream infections. Arch Intern Med.

[17] Gormley GJ, Fenwick T (2016). Learning to manage complexity through simulation: students’ challenges and possible strategies. Perspect Med Educ.

[18] Eost-Telling C, Kingston P, Taylor L, Emmerson L (2021). Ageing simulation in health and social care education: A mixed methods systematic review. J Adv Nurs.

[19] McLaughlin C, Barry W, Barin E, Kysh L, Auerbach MA, Upperman JS, Burd RS, Jensen AR (2019). Multidisciplinary simulation-based team training for trauma resuscitation: a scoping review. J Surg Educ.

[20] Murray RE, Ryan PB, Reisinger SJ. Design and validation of a data simulation model for longitudinal healthcare data. InAMIA Annual Symposium Proceedings 2011. Am Med Info Assoc. 2011:1176. PMC3243118 22195178

[21] What is ethics?. Government of Canada; 2015 [cited 2024 Feb 24]. Available from: https://www.canada.ca/en/treasury-board-secretariat/services/values-ethics/code/what-is-ethics.html

[22] Watts PI, Rossler K, Bowler F, Miller C, Charnetski M, Decker S, Molloy MA, Persico L, McMahon E, McDermott D, Hallmark B (2021). Onward and upward: introducing the healthcare simulation standards of best PracticeTM. Clin Simul Nurs.

[23] Diaz-Navarro C, Laws-Chapman C, Moneypenny M, Purva M. The ASPiH Standards - 2023: guiding simulation-based practice in health and care [cited 2024 Feb 25]. Available from https://aspih.org.uk

[24] Lewis KL, Bohnert CA, Gammon WL, Hölzer H, Lyman L, Smith C, Thompson TM, Wallace A, Gliva-McConvey G (2017). The association of standardized patient educators (ASPE) standards of best practice (SOBP). Adv Simul.

[25] Healthcare Simulationist Code of Ethics, [cited 2024 Feb 25]. Available from https://www.ssih.org/SSH-Resources/Code-of-Ethics

[26] Purdy E, Symon B, Marks RE, Speirs C, Brazil V (2023). Exploring equity, diversity, and inclusion in a simulation program using the SIM-EDI tool: the impact of a reflexive tool for simulation educators. Adv Simul.

[27] NaynaSchwerdtle P, Horton G, Kent F, Walker L, McLean M (2020). Education for sustainable healthcare: a transdisciplinary approach to transversal environmental threats. Med Teach.

